# Exploring the cessation process from adolescence to young adulthood in individuals with lived experience of nonsuicidal self‐injury: a qualitative study

**DOI:** 10.1002/jad.12417

**Published:** 2024-10-02

**Authors:** H. Andersson, L. Korhonen, K. Holmqvist Larsson, B. M. Gustafsson, M. Zetterqvist

**Affiliations:** ^1^ Center for Social and Affective Neuroscience, Department of Biomedical and Clinical Sciences Linköping University Linköping Sweden; ^2^ Department of Child and Adolescent Psychiatry Linköping Region Östergötland Sweden; ^3^ Barnafrid, Swedish National Center on Violence Against Children, Department of Biomedical and Clinical Sciences Linköping University Linköping Sweden; ^4^ Department of Psychiatry and Rehabilitation Psychiatric Clinic, Högland Hospital Region Jönköping Sweden; ^5^ CHILD research environment Jönköping University Jönköping Sweden

**Keywords:** Benefits and Barriers Model, cessation, nonsuicidal self‐injury, recovery, thematic analysis

## Abstract

**Introduction:**

Nonsuicidal self‐injury (NSSI) is a common and concerning behavior in adolescents. However, most adolescents cease NSSI as they transition into adulthood. Increased knowledge of the cessation process is needed. This study aimed to qualitatively explore the factors contributing to NSSI cessation in individuals with lived experience of NSSI, providing valuable insights for treatment strategies.

**Methods:**

Twenty‐six individuals assigned female sex at birth, between ages 20–22 years, from Sweden were interviewed between 2021 and 2023 in Linköping, Sweden. Of these, 21 individuals perceived themselves as having ceased NSSI and were included in the analysis. Thematic analysis and Hooley and Franklins' Benefits and Barriers Model of NSSI were used to analyze the transcripts.

**Results:**

Three overarching themes were generated: “Something inside me changed”, “Something in my close relationships changed”, and “Something in my life context changed”. The cessation of NSSI was associated with several key factors. Improved well‐being and envisioning a different future were pivotal in initiating the cessation process. Additionally, interpersonal relationships and support from others were interpreted as powerful motivators for change. Transitioning to a new social context and leaving behind a destructive environment provided opportunities for personal growth and enhanced well‐being, interpreted as initiators in the participants' broader life context.

**Conclusion:**

This study underscores the complexity of the NSSI cessation process and highlights the need for a comprehensive understanding of the underlying factors. Access to emotion regulation skills was perceived as a significant barrier to NSSI engagement. Clinical implications and different interventions to support NSSI cessation are discussed.

## 1 INTRODUCTION

Nonsuicidal self‐injury (NSSI), defined as the deliberate and direct destruction of body tissue without suicidal intent (Nock & Favazza, 2009), is a prevalent and concerning behavior among adolescents. Estimates suggest that around 17% of adolescents in the community have engaged in NSSI (Muehlenkamp et al., 2012; Swannell et al., 2014), with checklist measurements indicating even higher rates of 30–40% having tried the behavior at least once (Aspeqvist et al., 2024; Zetterqvist et al., 2013). In clinical samples, the prevalence of NSSI varies. However, it is higher than in community samples (Muehlenkamp & Tillotson, 2024), with up to 80% of adolescents in inpatient settings reporting lifetime NSSI (Hauber et al., 2019). NSSI peaks in mid‐adolescence and typically decreases as individuals transition to adulthood (Cipriano et al., 2017; Plener et al., 2015).

Although not defined as a suicidal behavior, NSSI is associated with an increased risk of suicidality in young adults (Kiekens et al., 2018). Additionally, NSSI is linked to long‐term negative consequences, such as increased symptoms of depression and anxiety (Daukantaitė et al., 2021). These adverse outcomes highlight the need for increased knowledge and understanding of the factors that contribute to the cessation of NSSI.

The cessation of NSSI is typically defined as a reduction or absence of NSSI engagement over a specified period of time. This concept is operationalized in various ways, such as reporting no NSSI incidents in the past year (e.g., Gray et al., 2022; Horgan & Martin, 2016; Whitlock et al., 2015), noting changes between different time points (e.g., Kiekens et al., 2017; Tatnell et al., 2014), or engaging in NSSI on fewer than 5 days in the past year, thereby no longer meeting the criteria for the NSSI disorder that was proposed in the fifth version of the Diagnostic and Statistical Manual for Mental Disorders in 2013 (American Psychiatric Association & Association, 2013). NSSI cessation is usually distinguished from recovery. While cessation can be a part of the recovery process, recovery is a more comprehensive concept. According to the person‐centered recovery framework (Lewis & Hasking, 2021), recovery reflects a broader spectrum of well‐being and resilience‐building. The framework, developed by Hasking and Lewis, involves realistic expectations and setbacks, normalizing thoughts and urges, fostering self‐efficacy, identifying strengths, finding alternatives to NSSI, addressing underlying adversities, accepting and addressing scarring, navigating disclosures, and achieving self‐acceptance. Furthermore, it is important to consider individuals' own perceptions of whether they have ceased NSSI. These perceptions may not always align with behavioral cessation and might be more indicative of recovery than mere behavioral cessation (Clareus et al., 2023).

Previous theoretical work on NSSI has primarily focused on understanding why individuals initiate and maintain NSSI (see, e.g., Hasking et al., 2017; Nock, 2010). The Benefits and Barriers Model (Hooley & Franklin, 2018) outlines the factors determining why an individual starts and continues engaging in NSSI. The model suggests that NSSI has potent benefits that are accessible to most people. According to the model, the benefits of NSSI are that it improves affect (and thus serves as an emotion‐regulatory function), gratifies self‐punishment desires, provides peer group affiliation, and can communicate distress or strength. These benefits have also been described as intrapersonal and interpersonal functions of NSSI (Klonsky & Glenn, 2009), with affect regulation and self‐punishment being especially common intrapersonal functions of NSSI (Klonsky, 2009). However, according to the Benefits and Barriers Model, most people do not access these benefits of NSSI due to powerful barriers that motivate them to avoid self‐injury. The suggested barriers to NSSI are a lack of awareness about NSSI, a positive view of the self, wanting to avoid physical pain, aversion to NSSI stimuli, and social norms. The model articulates that cessation of NSSI can be facilitated by rebuilding and strengthening these barriers.

Supporting the Benefits and Barriers Model, a meta‐analysis (Mumme et al., 2017) showed that both interpersonal (e.g., social connectedness and family support) and intrapersonal (e.g., emotion regulation, self‐esteem, and personal motives) factors facilitate NSSI cessation, underscoring the role of positive self‐image as a potential barrier to NSSI. In cross‐sectional studies comparing individuals who retrospectively reported having abstained from NSSI for at least 1 year, with individuals who had engaged in NSSI during the last year, NSSI abstinence was associated with less self‐blame (Horgan & Martin, 2016), less psychological distress and difficulties regulating emotions (Gray et al., 2022; Whitlock et al., 2015), increased self‐efficacy to resist engaging in NSSI (Claréus et al., 2023; Gray et al., 2022), distress tolerance, and positive emotional intensity but not decreased negative emotional intensity (Horgan & Martin, 2016). Furthermore, a longitudinal study (Tatnell et al., 2014) showed that individuals who had ceased NSSI for more than 1 year exhibited higher self‐esteem and perceived social support than those who had engaged in NSSI during the previous year. These findings suggest that improvements in social settings and support, as well as personal growth, are important factors in ceasing NSSI. However, as previously mentioned, cessation of NSSI does not always equal recovery (Lewis et al., 2019). For instance, Turner et al. (2022) found that NSSI cessation was associated with an increase in past‐year heavy drinking, cannabis use, and tobacco use in a longitudinal study. Sustained NSSI cessation, however, was associated with reductions in heavy drinking and tobacco use, as well as improvements in current depression, anxiety, externalizing symptoms, and peer victimization. These results support the idea of recovery as a nonlinear process (Lewis & Hasking, 2021), where cessation is an important step, but not the only factor contributing to recovery.

One of these steps is gaining access to alternative strategies (Lewis & Hasking, 2021). Since emotion regulation is the most common reason why an individual engages in NSSI (Klonsky, 2009; Taylor et al., 2018), and is described as a major benefit of NSSI in the Benefits and Barriers Model (Hooley & Franklin, 2018), emotion regulation strategies are of particular interest in both NSSI cessation and recovery. Here, emotion regulation is conceptualized as the strategies used to influence the experience, intensity, and expression of emotions (Gross & Jazaieri, 2014) and includes the awareness, understanding, and acceptance of emotional responses, the ability to engage in goal‐directed behaviors while inhibiting impulsive behaviors, using appropriate behaviors to modulate emotional responses, and willingness to experience negative emotions to pursue meaningful activities (Gratz & Roemer, 2004).

Perceived emotion regulatory capability, measured by the “limited access to emotion regulation strategies” subscale of the Difficulties with Emotion Regulation Scale (DERS; Gratz & Roemer, 2004), has been found to predict NSSI cessation (Kiekens et al., 2017). Furthermore, Kiekens et al. (2017) found that belief in one's emotion‐regulatory ability mediated the relationship between perceived social support, academic stress, life satisfaction, and NSSI. Emotion regulation difficulties have also been found to mediate NSSI reduction in treatments focused on increasing emotion regulation strategies in adolescents (Bjureberg et al., 2018; Bjureberg et al., 2023) and adults (Gratz et al., 2012; Gratz et al., 2015). Taken together, this strengthens the empirical support for the importance of emotion regulation skills in the NSSI cessation process.

Qualitative studies have broadly explored the process of NSSI cessation in clinical and community samples of adolescents and young adults with lived experience of NSSI. The cessation process has been described as highly individual (Stänicke et al., 2020), involving various internal and external factors (Limsuwan et al., 2023). Important relationships, life circumstances, coping skills, and treatment have been reported to be significant factors in the cessation process (Gelinas & Wright, 2013; Hansson et al., 2020; Limsuwan et al., 2023; Rissanen et al., 2013; Stänicke et al., 2020; Whitlock et al., 2015). Claréus et al. (2021) described the process as involving turning points that lead to increased agency, viewed as essential for personal growth and change. Hambleton et al. (2022) further found that the process of ceasing NSSI often involved multiple reasons for discontinuation in 100 individuals who engaged in NSSI during adolescence. Among the most common reasons cited were changes in interpersonal relationships and receiving professional treatment. Here, we add to, and expand on, the existing literature on why individuals cease NSSI by exploring the cessation process in individuals with lived experience through the lens of the Benefits and Barriers Model.

### 1.1 Aim

The current study had two main aims. The first was to explore the cessation process of NSSI in individuals with lived experience of NSSI, with a particular focus on emotion regulation. Based on these results, the second aim was to analyze the process of NSSI cessation using the Benefits and Barriers Model framework (Hooley & Franklin, 2018). The study focused on the following research questions:

‐ How did participants describe what initiated their NSSI cessation process, and why did they no longer engage in NSSI?
‐ What benefits and barriers of NSSI can be interpreted from the participants' descriptions of their NSSI cessation process?

## 2 MATERIALS AND METHODS

This study is part of a follow‐up study of neurobiological markers of NSSI (Mayo et al., 2021; Perini et al., 2019).

### 2.1 Participants

Thirty adolescents (aged 15–17, *M* = 15.9, SD = 0.8) with ongoing NSSI were recruited from child and adolescent psychiatry (CAP) in Sweden between June 2016 and March 2018 as part of a study that examined neurobiological markers of NSSI (Mayo et al., 2021; Perini et al., 2019). Inclusion criteria for participants required having engaged in five or more instances of NSSI during the past 6 months irrespective of psychiatric diagnosis and being a female between 15 and 18 years old. The exclusion criteria included current or lifetime diagnosis of schizophrenia, bipolar or psychotic disorder, alcohol/drug dependence, and IQ below 80. See Mayo et al. (2021) and Perini et al. (2019) for further details on the demographic characteristics of the original study sample.

During the follow‐up study, which was conducted between 2021 and 2023, the 30 young adults were contacted again. Of the original 30 participants, one participant declined to participate in the follow‐up, one could not be reached, and two participants provided oral consent in the initial contact but did not provide written informed consent, and thus were omitted. In total, 26 young adults (86.7% of the original sample, 100% assigned female sex at birth) between ages 20–22 years (*M* = 21.2, SD = 0.8) were included and interviewed. Of these, 19 (73.1%) had ceased NSSI more than 1 year ago, two (7.7%) had their latest NSSI episode 6‐8 months ago, and five (19.2%) had ongoing NSSI. The current analysis included the 21 participants who perceived themselves as having ceased NSSI with no current NSSI. Their mean age was 21.2 (SD = 0.8) years. The mean age for NSSI cessation was 17.6 (SD = 1.4) years. See Table 1 for further details on the demographic characteristics of participants.

**Table 1:** Participant demographics, *N* = 21.

Demographic characteristics	Total sample, *n* (%)
Sex	
Assigned female sex at birth	21 (100.0)
Age *m* (sd)	21.2 (0.8)
Occupational status	
Working 75–100%	5 (23.8)
Working 25–74%	4 (19.0)
University/college studies 100%	5 (23.8)
Other studies 75–100%	3 (14.3)
Other studies ≤50%	1 (4.8)
Other^a^	3 (13.4)
Healthcare contact	
Psychiatric contact	7 (33.3)
Primary care contact	1 (4.8)
Waiting for psychiatric assessment/treatment	2 (9.5)
No ongoing healthcare contact	11 (52.4)
Psychiatric diagnoses^b^	
Diagnosis	17 (81.0)
No diagnosis	4 (19.0)
Living arrangements	
Single households	4 (19.0)
Living with partner	10 (47.6)
Living with parents	5 (23.8)
Other living arrangements	2 (9.5)

^a^ For example unemployment or sick leave.

^b^ Psychiatric diagnoses included depression, autism (including traits), attention deficit disorder (ADD)/attention deficit hyperactivity disorder (ADHD; including unspecified), posttraumatic stress disorder, eating disorder (including unspecified), bipolar disorder, anxiety disorder (including unspecified), and borderline personality disorder (including traits). The most common diagnoses were ADD/ADHD, anxiety disorder, and depression.

### 2.2 Data collection

Participants were initially contacted by telephone and provided information about the follow‐up study. Subsequently, written information was sent to them by letter. After reading the written information, participants were contacted by telephone again and gave oral consent to participate. Written informed consent was obtained from participants before the assessment and interview session at the hospital. For participants who lived far away, we offered the option of a digital interview via Skype or Zoom. One participant was interviewed digitally, and the rest were interviewed face‐to‐face. For the informant who chose the digital option, we sent the informed consent in advance, which was then signed and returned by the participant by post. In addition to the interview, participants completed self‐report questionnaires, and we assessed psychiatric symptoms and diagnoses. The session began with the interview, followed by the completion of questionnaires and assessments. The interviews were conducted between November 2021 and February 2023.

### 2.3 Interview

Two licensed female psychologists (HA, MZ) with experience in clinical work and research on NSSI and clinical child and adolescent psychiatry conducted the interviews. MZ, one of the interviewers, had previously been involved in the assessment procedure of the original study 5 years ago and had met the participants in this capacity but did not have any ongoing clinical relationship with them. The interviewers presented the aim of the study at the beginning of the interview. The semi‐structured interviews included open questions that focused on three areas: the process of NSSI cessation, the benefits and barriers of NSSI, and participants' experiences of healthcare and treatment of NSSI. Prompts were used to obtain more details regarding the participants' experiences. The interviews had a mean length of 25 min (varying between 10 and 40 min) and were audio recorded and transcribed verbatim.

### 2.4 Ethical considerations

The procedure and study received approval from the Regional Ethical Review Board of Linköping and the Swedish Ethical Review Authority (2015/273‐31; 2016/224‐32; 2021‐04328). All study participants provided written informed consent. Personally identifiable information has been anonymized and removed, and pseudonyms are used when presenting quotes.

### 2.5 Data analysis

Data were analyzed with reflexive thematic analysis to generate themes with shared meaning. Following Braun and Clarke's six‐step process (Braun & Clarke, 2006, 2019, 2022), the analysis included: familiarizing oneself with the data, generating initial codes, searching for themes, reviewing themes, defining and naming themes, and producing the report. MZ and HA conducted the interviews, and HA transcribed some of the interviews as part of the familiarization process (Step 1). The remaining interviews were transcribed by a research assistant. All personal information, such as names of people or specific places, was removed from the transcripts and they were imported into NVivo version 20 for Windows. HA, MZ, KHL, and BMG read and reread the transcripts and discussed them jointly to familiarize with the material. In Steps 2, 3, and 4, HA coded the data, collated the codes into potential themes, and reviewed and altered them by checking their correspondence with the codes and excerpts. The analysis process moved back and forth between the six steps, and ongoing discussions were held between all authors regarding potential themes, defining themes, and naming themes (Steps 4 and 5). Lastly, HA wrote the report (Step 6).

The analysis was conducted in accordance with a critical realist approach, which acknowledges the reality of the participants but recognizes that the knowledge of reality is produced by social structures (Fletcher, 2017). We employed both inductive and deductive approaches in the analysis, consistently analyzing the entire data set throughout the process of analysis. The analysis began inductively with a detailed exploration of participants' descriptions of their cessation processes. Early on in the transcript review, we perceived that references to emotion regulation recurred in the participants' descriptions. This led us to focus on this aspect in subsequent stages of the analysis. Initially, coding was conducted at the semantic level, but as the analysis progressed, it evolved into a latent analysis with a concentrated focus on emotion regulation. After generating initial themes, we utilized the Benefits and Barriers Model (Hooley & Franklin, 2018) to interpret the findings related to the cessation process.

All authors/researchers involved in the analysis have clinical backgrounds: HA and MZ are psychologists, KHL is a psychotherapist, LK is a child psychiatrist, and BG is a nurse. All authors have experience working with children, adolescents, and their parents (including individuals with NSSI) in different settings (child and adolescent psychiatry, primary care, and schools). The authors also have experience with various treatment approaches, such as cognitive behavioral therapy (CBT), acceptance and commitment therapy, emotion regulation skills training, and dialectical behavioral therapy, as well as qualitative research. All authors are female.

## 3 RESULTS

Participants reported experiencing both benefits of and barriers to NSSI before starting the cessation process. Participants described emotion regulation as the most prominent benefit of NSSI. Other benefits included self‐punishment and communicating distress. Social norms were interpreted as a common barrier to NSSI, which required significant effort for participants to overcome (e.g., concealing physical signs of NSSI). This analysis focused on the factors that determined a shift towards NSSI cessation (hereafter referred to as initiators), and participants' reasons why they no longer engaged in NSSI, interpreted as barriers to NSSI engagement (see Table 2). Additionally, the behaviors the participants adopted to replace NSSI were analyzed (referred to as replacement behaviors in Table 2).

**Table 2:** Themes and subthemes, initiators of change, barriers to NSSI, and replacement behaviors to access the benefits of previous NSSI in the cessation process.

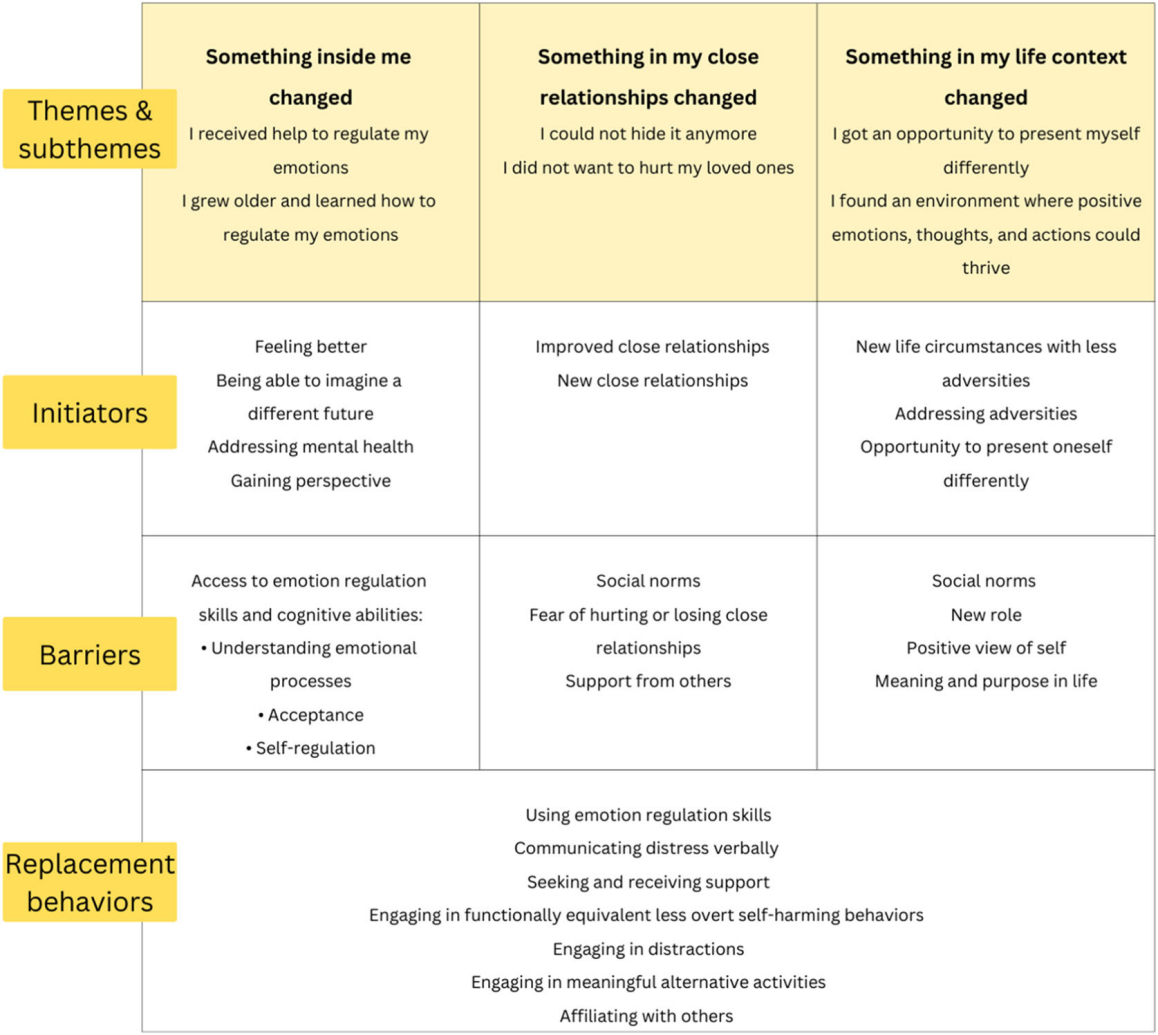

**Figure 1:**
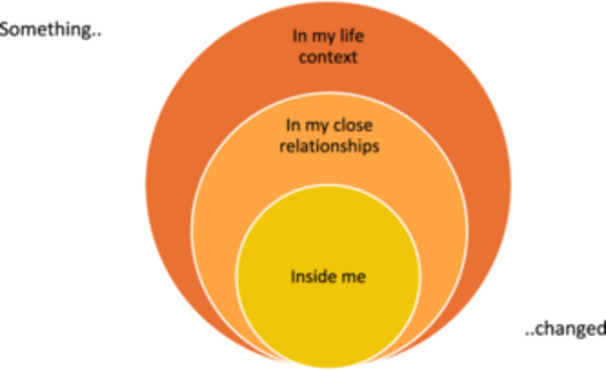
Overarching Themes in the NSSI Cessation Process.

The analysis of the cessation process generated three overarching themes (Figure 1). The first theme, “Something inside me changed”, describes participants' descriptions of NSSI as a way of dealing with emotional difficulties and how internal changes initiated the cessation process. The second theme, “Something in my close relationships changed”, describes a positive change in participants' close relationships, and concerns that NSSI would negatively affect these relationships initiated the process of NSSI cessation. The third theme, “Something in my life context changed”, describes how changes in the participants' life circumstances gave rise to opportunities for personal growth, which were considered the main reason for NSSI cessation. Participants described a reciprocal relationship between these three overarching themes, where changes in one theme often led to changes in the others.

### 3.1 Theme 1. Something inside me changed

This theme captures participants' perceptions of emotional difficulties (e.g., the intensity or duration of emotions) as the main reason for NSSI engagement, emotion regulation as a benefit of NSSI, and their perceptions that something inside them changed, making them feel better and/or being able to image a different future. *Feeling better* and *being able to imagine a different future* were interpreted as the initiators to cease NSSI (see Table 2). The main barriers to engaging in NSSI were the gained *access to emotion regulation skills* and *cognitive abilities*. The internal changes were also described as affecting the whole life situation of the participants. For instance, when participants felt better, they more frequently opened up to the importance of their loved ones, which also helped them maintain NSSI cessation and vice versa.

#### 3.1.1 Subtheme—I received help to regulate my emotions

In this subtheme, participants described internal challenges in coping with their emotions and mainly attributed their NSSI cessation to different professional interventions that they participated in that targeted their well‐being. Some participants emphasized the importance of taking medication in this process. Using medication was interpreted as alleviating participants' low mood, making them feel better, stabilizing their emotions, reducing anxiety or the intensity of their emotions, or calming down, which they described as a major contributing factor in not needing NSSI anymore. *Addressing underlying mental health problems* was thus interpreted as initiating the cessation process. The emotional experience was portrayed as the problem, as it was seen as the reason for the NSSI engagement and the problem they needed to solve. Charlie described “…with medication, I stayed very even, I could never be really happy, but I couldn't be really sad… and then I never got to those deepest moments.” Some individuals attributed their improved emotion regulation skills to the psychotherapy they participated in. Kim, for instance, explained “Both the emotion regulation course and [the CBT], I think was the reason why I managed to stop… because I could put into words and understand what was happening inside me, it wasn't just chaos…”.

#### 3.1.2 Subtheme—I grew older and learned how to regulate my emotions

The essence of this subtheme is a natural process of developing, maturing, and feeling better over time. The key initiators were interpreted as *feeling better, being able to imagine a different future*, and *getting perspective* on their situation, which contributed to ceasing NSSI. Jessica, for example, described the process of ceasing NSSI as not needing the physical pain anymore, as she did not experience the same amount of emotional pain. “I think it was that the emotions weren't as strong anymore… that physical pain actually… was needed.”

Participants also reported that they learned how to regulate their emotions from past emotionally challenging situations and life experiences. Participants expressed increased *understanding of their emotions, skills in identifying, accepting*, and *expressing emotions*, as well as *enduring* and *distracting themselves* from intense emotions. Thus, they reduced the need for NSSI due to internal processes of *improved abilities to self‐regulate* over time. Some expressed a communication benefit of NSSI, but could, as time passed, replace this behavior with functionally equivalent behaviors, such as *communicating their emotions and needs verbally*. Thus, how they dealt with their emotions was portrayed as what needed to change to cease NSSI.

I think I've learned to deal with my emotions better… I don't really have anything specific that I do, but I just know that, because it was also about that age… it was very hard to deal with emotions and it was difficult to know how to go about it, but now I have found some things that do work… you've got to know yourself a bit better and grown up a bit. I know that if I feel like this I should avoid this and that, or do it this way instead. [Billie]

### 3.2 Theme 2. Something in my close relationships changed

This theme captures participants' expressions of experiencing *a positive change in their close relationships* as the initiator of their NSSI cessation process (see Table 2). The participants described that their relationships improved and became more important as they began to feel better, and vice versa. NSSI was interpreted as a risk for harming these relationships. Positive close relationships became an initiator for change. Participants described increased *support and help* from their loved ones when dealing with challenging situations and emotions, which was interpreted as being a barrier to NSSI.

#### 3.2.1 Subtheme—I could not hide it anymore

This subtheme highlights the perceptions of NSSI as a shameful activity that could no longer be concealed in a new interpersonal context, which contributed to participants ceasing NSSI. The perceived social norms of NSSI and the shame of NSSI engagement were frequently reported in participants' accounts for ceasing NSSI. They described how self‐criticism and shame triggered NSSI, but also feeling shame over NSSI engagement, and that this, from the very beginning, was experienced as something wrong. “It's kind of shameful. It's not accepted to do this. It's wrong.” [Debbie]. Participants dealt with the shame and overcame the barrier of social norms by hiding bodily signs of NSSI. Still, for some participants, a new interpersonal context meant that they could no longer conceal their NSSI engagement. For instance, when starting a new romantic relationship that meant a lot to them, or when parents found out, participants had to navigate disclosure, which often initiated the cessation process*. Social norms* were thus interpreted as a barrier to NSSI engagement. Mika described, “…I didn't want to… hurt myself when… he could see, like, when you sleep together and… it can be seen… he would have seen right away.”

#### 3.2.2 Subtheme—I did not want to hurt my loved ones

Some participants stated that they stopped engaging in NSSI due to concerns about its impact on their loved ones. They described a positive change in their relationships with parents, friends, or partners and did not want to risk harming or jeopardizing those relationships. In some cases, participants described the experience of witnessing harm to loved ones as painful and expressed a desire to stop engaging in NSSI to prevent further harm.

Since my relationship with my parents had got better, I didn't want to continue [with NSSI] so as not to feel that they would be disappointed because they had been there to help me, so then I was like “No, it's enough now, I have to turn the page”. [Sara]

Some participants compared the pros and cons of engaging in NSSI. They noted that the negative consequences of hurting loved ones outweighed the potential benefits of NSSI, which contributed to NSSI cessation. *Fear of hurting loved ones* was thus interpreted as a barrier, and for some meant ceasing NSSI but replacing NSSI with *other self‐destructive behaviors*.

But I had started replacing it [NSSI] with alcohol a bit…somewhere I realized that I don't need to hurt myself in the form of scarring my body, but that I could find other ways instead that were less visible, that no one would notice… Because I knew that it was really tough on my parents when they realized… that I was doing it and how bad I felt. So I didn't want to show them that I was still feeling bad, because they thought it was better. [Dylan]

### 3.3 Theme 3. Something in my life context changed

Participants described the positive impacts of a *modified environment* on emotional experiences, as well as providing opportunities for growth. Being in a harmful environment was described as triggering negative emotions, which participants expressed that they had earlier coped with through NSSI engagement. *Leaving that environment*, *addressing adversities*, and having an *opportunity to present oneself differently* were interpreted as significant factors for participants in initiating the NSSI cessation process (see Table 2). A new context was interpreted as affecting general well‐being in participants through different *social norms*, being in a *new role, finding meaning and purpose in life*, and *getting a more positive view of oneself*, which were analyzed as barriers to NSSI.

#### 3.3.1 Subtheme—I got an opportunity to present myself differently

Participants described how being in a new social context with different *social norms* became a barrier to NSSI. Participants expressed concerns about how others would perceive them in a new social context and fears of being perceived as vulnerable and emotionally unstable if they continued with NSSI. Marge, for instance, said: “I didn't want people to see that I was vulnerable or that I was doing that [engaging in NSSI]”. In a new context, participants had the *opportunity to present themselves differently*, which was therefore interpreted as an initiator for change. In the new social context, participants started to question their NSSI engagement, reflect on how the social context affected them, and how they dealt with their emotions.

I think I started to question why I cut myself… and was in a different social context than when I started and in the new social context it wasn't the normal way… to deal with your emotions… I think it had previously been an environment in which I perhaps did not feel so good… and there were several others who did not feel good… and to deal with it by… doing things that are not so good for you was quite normal and accepted. [Kim]

#### 3.3.2 Subtheme ‐ I found an environment where positive emotions, thoughts, and actions could thrive

Some participants described that a new context meant that they found new meaning and purpose in life. They perceived themselves to be in the right place with *people and activities they enjoyed* and could imagine a future for themselves, which increased their well‐being, and reduced the need for NSSI.

It was during the time I was in high school, I got along very well with some of the students… for the most part, I felt that I had found my place, that this was what I wanted to do… I wanted to work in healthcare. [Francis]

A changed social context was also perceived by some participants as influencing the way they *viewed themselves*, which became a barrier to engaging in NSSI. Cassandra described, “It was probably that I got a greater sense of self‐worth… and a better relationship with my body… that I got a lot of encouragement and support when I danced… and then I didn't want to destroy that body.” Participants also described a more positive view of themselves due to NSSI cessation.

## 4 DISCUSSION

This qualitative study explored the cessation process in 21 young adults with lived experience of NSSI, focusing on what initiated this process. The findings were interpreted through the lens of the Benefits and Barriers Model (Hooley & Franklin, 2018). Three overarching themes were generated from the analysis: “Something inside me changed”, “Something in my close relationships changed”, and “Something in my life context changed”. Improvements in these intrapersonal, interpersonal, and life contextual areas, mutually influenced each other. Participants described multiple initiators of change, with changes in one area often leading to changes in the others. The results of the current study highlight the significance of individual growth in the cessation of NSSI, with enhanced emotion regulation skills and cognitive abilities serving as barriers to continued engagement in NSSI within the intrapersonal theme. Furthermore, improved and new close relationships were reflected as strong motivators for change, interpreted as playing a pivotal role in initiating the cessation process. Finally, the transition to a new social context and leaving a destructive environment behind was perceived as an opportunity to present oneself differently, while simultaneously fostering future growth and well‐being. Throughout the themes, NSSI was viewed as serving various universal needs. In the cessation process, participants engaged in functionally equivalent behaviors as replacement strategies to address these needs, ultimately reducing their reliance on NSSI and aiding in the maintenance of cessation.

### 4.1 Something inside me changed

A recurrent theme in participants' descriptions was the role of internal changes, including personal growth, in initiating the cessation of NSSI. Improved emotion regulation, cognitive abilities, and increased self‐knowledge were interpreted as important in this process. Personal growth is essential within the person‐centered framework of recovery (Lewis & Hasking, 2021). For instance, finding alternative strategies is described as an important component. This process requires time and effort, as no single alternative suits all. Developing emotion regulation skills and identifying alternative behaviors that serve the same function as NSSI can reduce the need for NSSI, increase self‐efficacy in resisting urges, and be critical in ceasing and maintaining cessation of NSSI (Lewis & Hasking, 2021). Participants in earlier studies (Gelinas & Wright, 2013; Kruzan & Whitlock, 2019; Whitlock et al., 2015) have similarly reported that improved emotion regulation and coping skills can reduce the need for NSSI and serve as reasons for cessation. Emotion regulation is frequently cited as the primary reason individuals engage in NSSI (Taylor et al., 2018) and is acknowledged as a benefit in the Benefits and Barriers Model (Hooley & Franklin, 2018). In the current study, participants identified several emotion regulation skills as important, including the ability to identify, label, and accept emotions. They also emphasized the importance of experiencing and managing emotionally intense situations, perceiving themselves as capable of handling these challenges. This aligns with the concept of “perceived emotion regulatory capability” as a critical factor in ceasing NSSI (Kiekens et al., 2017). This suggests that perceiving oneself to be competent to downregulate emotion during adversity, rather than experiencing low levels of emotional distress, might be crucial to successfully ceasing NSSI. The ability to resist urges is further described as important in the recovery process, even after ceasing NSSI, as thoughts and urges may persist (Lewis & Hasking, 2021). While access to emotion regulation skills is not described as a barrier to NSSI engagement in the Benefits and Barriers Model (Hooley & Franklin, 2018), the results of the current study emphasize its significance as a barrier to continuing NSSI engagement.

Individual growth, encompassing enhanced cognitive abilities, and emotion regulation skills, was interpreted as a barrier to NSSI engagement and outcomes of treatment, maturation, and experience. While these skills may develop naturally with maturity, they are not exclusively linked to age (Whitlock et al., 2015). Participants in the current study emphasized developing these skills and abilities through experiential learning, gradually learning how to accept difficult emotions and urges, and finding alternative activities, resulting in increased self‐knowledge. These skills were also described as gained through treatment and/or maturation. Some participants reported self‐efficacy in learning new skills and handling difficult situations, while others attributed their NSSI cessation to medication.

Perceptions of the effect of treatment for NSSI have been found to vary (Hansson et al., 2020; Rissanen et al., 2013), and were also indicated by the participants in this study. Pharmacological treatment and psychotherapy were mentioned as contributing to the cessation process by increasing general well‐being and gaining access to emotion regulation skills and cognitive abilities. This could potentially be interpreted in the context of the study setting. The current follow‐up study employed a clinical sample originally recruited from CAP, where a medical view of NSSI predominates, likely affecting participants' perceptions of which factors maintain NSSI and are necessary for cessation (e.g., emphasizing treatment, such as medication, in their cessation process). Notably, several participants did not mention treatment as contributing to the cessation process but rather emphasized nontherapeutic factors such as maturation, despite all having been in treatment at CAP. This suggests that other factors, such as cognitive and emotional development during adolescence, which are ongoing processes during this period (Steinberg, 2005), also play significant roles.

Several participants also perceived comorbid conditions, such as ADHD and depression, as primary reasons for their engagement in NSSI. Addressing these underlying issues was, for some of the participants, seen as the main initiator of NSSI cessation, as the need for NSSI diminished when these problems were treated. This highlights the importance of individualizing the focus in the treatment of NSSI, where some individuals perceive it necessary to address underlying psychiatric conditions (Andersson et al., 2024). Treatment of underlying mental health problems or an overall improvement in mental health is therefore critical for some individuals in the cessation of NSSI (Hambleton et al., 2022).

### 4.2 Something in my close relationships changed

Participants described how positive changes in their relationships initiated their cessation process. The beneficial effects of interpersonal relationships on NSSI cessation have been noted by participants in both community samples (Gelinas & Wright, 2013; Hambleton et al., 2022; Kruzan & Whitlock, 2019; Whitlock et al., 2015) and clinical samples (Hansson et al., 2020; Limsuwan et al., 2023). Several participants perceived closer relationships as improving their well‐being and providing the support they previously lacked. The importance of receiving social support in the cessation of NSSI has been documented in previous studies (Tatnell et al., 2014), with familial support being particularly emphasized (Mummé et al., 2017). Wadman et al. (2018) further describe the critical role parents play in adolescents' NSSI engagement. Parents can be perceived as an ongoing source of support but can also potentially trigger NSSI and impact future disclosures, depending on their response to adolescents' disclosure of NSSI. Judgmental, emotional, or trivializing reactions can lead adolescents to conceal their NSSI to avoid anticipated conflicts in the family (Wadman et al., 2018). The results of the current study expand on these findings by showing how familial reactions can affect participants' cessation process. For instance, the navigation of disclosure was evident in the participants' descriptions, where they described an anticipated risk of disrupting their close relationships if they continued to engage in NSSI. Social norms were thus interpreted as a common barrier to NSSI engagement. However, these norms only became a barrier when relationships grew closer. This highlights the central role of navigating disclosure in the active process of ceasing NSSI, a theme also addressed in the person‐centered framework of NSSI recovery (Lewis & Hasking, 2021). This process may be ongoing even after cessation, as scars may unintentionally disclose NSSI history.

The participants' descriptions were largely concerned with the avoidance of the aversive consequences of NSSI, including a desire to conceal scars and avoid becoming a burden to others. This was interpreted through a stigma framework, where the public stigmatization of NSSI was interpreted as internalized by the participants. Hiding and covering scars are described as common ways of managing the anticipated stigmatization of NSSI (Staniland et al., 2021) and anticipated stigmatization appeared to play an important role in the cessation of NSSI among the participants. This may have contributed to participants' focus on change, rather than acceptance, in their accounts for ceasing NSSI at the moment of the interviews. The increased shame and guilt accompanying the stigma of NSSI (Staniland et al., 2021) may further trigger the behavior, or trigger individuals to engage in other self‐destructive behaviors, as reported by some participants in the current study.

Participants emphasized, as did Wadman et al. (2018), the helpful role of close relationships, where the communication of distress is crucial. In relationships characterized by adversity or in the absence of close relationships where individuals are not encouraged to communicate distress verbally or seek help, or communication attempts are not taken seriously, NSSI can serve as a means of expressing distress, one of the benefits of NSSI described by the Benefits and Barriers Model (Hooley & Franklin, 2018). The results of the current study contribute information on the cessation process by highlighting the importance of close relationships that foster healthy ways of communicating distress.

### 4.3 Something in my life context changed

Participants also described how a new life context initiated the cessation process. Finding a positive environment where participants could engage in meaningful activities and find a purpose in life was perceived as significantly improving their well‐being. Identifying strengths, including meaningful hobbies and activities, is a key component of the person‐centered framework for NSSI recovery (Lewis & Hasking, 2021). For some participants, this was essential for envisioning a positive future and ceasing NSSI.

Discovering a new environment with meaningful activities and supportive friends improved self‐perception for some participants. Being encouraged in activities and receiving positive feedback boosted self‐confidence, which in turn fostered a desire to take care of oneself rather than engage in NSSI. This re‐establishment of a positive self‐view acted as a barrier to NSSI, confirming the Benefits and Barriers Model (Hooley & Franklin, 2018). However, the results of the current study extend the theory by suggesting a reciprocal relationship between positive self‐view and NSSI cessation. Participants reported that ceasing NSSI also contributed to an improved or more accepting self‐perception.

Several participants identified underlying adversities in their life context contributing to NSSI engagement. Addressing these adversities or leaving environments that triggered NSSI was described as crucial in the cessation process. Relief from destructive environments has been noted as important for cessation, enhancing individuals' agency and well‐being (Claréus et al., 2021). Anticipated behaviors of emotion regulation within a given context also play a vital role in NSSI engagement, and finding a new context with healthy expectations has been described as a turning point in the cessation process (Hansson et al., 2020). This was corroborated by participants in the current study, where the initiator of change was interpreted as finding a new social context where NSSI was no longer perceived as a socially acceptable way to handle emotions. Thus, social norms became a barrier to NSSI engagement and were repeatedly reported by participants, confirming the Benefits and Barriers Model (Hooley & Franklin, 2018).

Participants' descriptions of their NSSI cessation process were primarily characterized by the struggle to avoid association with negative stereotypes associated with NSSI. The avoidance was interpreted as a key factor in initiating the cessation process. Negative stereotypes, such as being manipulative (Lloyd et al., 2018), attention‐seeking (Klineberg et al., 2013; Long, 2018), or being blamed for their NSSI (Staniland et al., 2021), can become internalized and significantly influence how participants approach their NSSI and potential disclosures (Wadman et al., 2018). This study deepens the understanding of stigmatization's effects on the cessation process. Participants expressed a fear of being labeled in new social contexts, anticipating that such stigmatization could disrupt relationships or careers due to their NSSI history.

The stigmatization of NSSI has detrimental effects on individuals with lived experience of NSSI, exacerbating suffering. In a new context, participants perceived an opportunity to present themselves differently to avoid NSSI stigma, which was interpreted as a primary initiator of NSSI cessation. The evident self‐stigmatization in the participants' accounts highlights the nonlinear and ongoing process of recovery (Lewis & Hasking, 2021). Recovery is a gradual process, and the participants in the current study are still young with only a few years having passed since they engaged in NSSI. Over time, other aspects of recovery, such as accepting scars and oneself may become more prominent. This distinction emphasizes that cessation and recovery from NSSI are separate constructs, where ceasing NSSI is a step towards recovery but does not necessarily equate to full recovery (Lewis et al., 2019).

### 4.4 Reflexivity

To guide the reader in considering the transferability of these findings, it is essential to interpret the results within the context of the employed methodology. In this study, we aimed to explore how individuals with lived experience of NSSI perceive their cessation process, while we simultaneously acknowledge the social factors influencing the participants' views and our interpretations of their descriptions. A significant factor likely shaping the participants' perspectives is that all participants had received treatment during adolescence in a medical context where a medical model of NSSI prevailed, potentially focusing on symptom reduction rather than strength building. This medical model likely influenced participants' perceptions of the cessation process. Additionally, all participants were assigned female sex at birth, limiting the transferability of the results to other groups.

Several participants reported experiences indicative of enacted stigma, which likely shaped their views of NSSI and its cessation, as well as how they described their cessation process. This issue was addressed in the research group through discussions on how to represent the participants' perspectives without perpetuating the stigmatization of NSSI. Consequently, it is important to note that this study focused primarily on the cessation of NSSI. The recovery process was not explicitly explored in the interviews, limiting further interpretations regarding recovery. However, recovery‐related constructs were reflected upon in the interviews and were evident to varying degrees in participants' narratives. To present individuals' perspectives ethically and respectfully, a person‐centered framework (Hasking et al., 2024) was employed during the interviews, the analysis, and the writing process.

The research group's background was also continuously considered to understand how it influenced the research process. Specifically, the clinical experience with children and adolescents, including those affected by NSSI, informed the analysis, adding depth to the interpretation of the data.

### 4.5 Implications

The findings of the current study on the cessation process of NSSI in individuals with lived experience have important implications for clinicians. Addressing the stigmatization of NSSI is crucial, as excessive emphasis on reducing NSSI may amplify feelings of shame further. Therefore, clinicians need to approach NSSI in a validating and understanding manner. Another way of approaching the stigmatization of NSSI is through educating the individuals around adolescents, such as teachers, parents, but also adolescents themselves.

Furthermore, it is pivotal to recognize the individuality and complexity involved in ceasing NSSI. In treatment, it is essential to tailor the understanding of NSSI functions to each person, considering individual, family, friends, and life context as potential factors that initiate and maintain NSSI engagement. This comprehensive approach is vital for effective conceptualization and to tailor subsequent interventions. For instance, interventions may include teaching emotion regulation skills to parents of adolescents with NSSI, as well as addressing adversities in the life context, such as destructive relationships or bullying. All levels of influence must be considered to support individual progress. Emphasis should be placed on creating or finding new environments that promote personal growth, such as those fostering emotion regulation skills. Additionally, nontherapeutic factors need to be considered when addressing cessation of NSSI. While targeting emotion regulation skills can benefit some individuals, it is essential to address whether these skills can be effectively applied within their specific contexts, not merely possessed as knowledge. Both being able to use the skills practiced in therapy, but also that these skills are being reinforced in the individual's life context.

Focusing solely on reducing NSSI can have adverse effects, as this can be perceived as invalidating, with individuals not viewing NSSI as the primary issue requiring treatment. Some individuals may perceive their main problem as something else, such as neurodevelopmental disorders or adverse life circumstances, that they think is more appropriate to target. In other cases, there is a need to prioritize values in life, and find meaning and purpose for individuals to engage in other activities, increasing well‐being and motivation. Some participants in the current study reported replacing NSSI with other harmful functionally equivalent behaviors. This substitution may reflect an unintended consequence of an excessive focus by others on reducing NSSI, potentially leading individuals to adopt alternative harmful behaviors to handle underlying issues. Therefore, the cessation of NSSI should not be equated with recovery, as individuals may continue to struggle with challenges that need to be addressed.

Close relationships have an important impact on NSSI cessation. Enhancing social skills can help individuals improve existing relationships or build new ones, contributing to their overall support system. Finding contexts in which social relationships can be created is also necessary.

NSSI cessation can be a multifaceted process, with several contributing factors, and this process can take time. Clinicians need to recognize that not all factors maintaining NSSI can be addressed in individual therapy. Collaboration with other community agencies, such as schools, can be beneficial in targeting contextual factors. For example, teaching and supervising teachers and school personnel to address unhealthy social norms and stigma, create healthy approaches to emotion regulation, and address bullying can be effective strategies.

## 5 CONCLUSION

In this study, 21 young adults with lived experience of NSSI reflected on their cessation process. Participants described the cessation process as initiated by intrapersonal, interpersonal, and contextual factors. Although improved emotion regulation was not always described as initiating the cessation process, changes at the interpersonal or contextual levels often had a positive effect on emotion regulation skills, such as expressing emotions verbally and seeking help when needed. Taken together, the cessation process can be conceptualized as individual and multifaceted, occurring over time, and involving reciprocal changes at intrapersonal, interpersonal, and contextual levels.

## AUTHOR CONTRIBUTIONS

**H. Andersson**: Conceptualization, Formal analysis, Investigation, Methodology, Visualization, Writing—Original draft **L. Korhonen**: Supervision, Writing—Review and Editing **K. Holmqvist Larsson**: Formal analysis, Writing—Review and Editing **B. M. Gustafsson**: Formal analysis, Supervision, Writing—Review and Editing **M. Zetterqvist**: Conceptualization, Formal analysis, Funding acquisition, Investigation, Methodology, Supervision, Writing—Review and Editing.

## CONFLICT OF INTEREST STATEMENT

The authors declare no conflict of interest.

## ETHICS APPROVAL AND CONSENT TO PARTICIPATE

The study was approved by the Regional Ethical Review Board of Linköping and the Swedish Ethical Review Authority (2015/273‐31; 2016/224‐32; 2021‐04328). All participants signed an informed consent form.

## CONSENT FOR PUBLICATION

Not applicable.

## Data Availability

Data are not available since we do not have participants' or ethical permission to share data.
